# Complete mitochondrial genome sequence of *Empoascanara dwalata* (Hemiptera: Cicadellidae: Typhlocybinae)

**DOI:** 10.1080/23802359.2020.1772141

**Published:** 2020-06-01

**Authors:** Xiaoxiao Chen, Zhouwei Yuan, Can Li, Yuehua Song

**Affiliations:** a School of Karst Science, Guizhou Normal University/State Engineering Technology Institute for Karst Desertification Control, Guiyang, China; b Guizhou Provincial Key Laboratory for Rare Animal and Economic Insect of the Mountainous Region, Guiyang University, Guiyang, China

**Keywords:** *Empoascanara dwalata*, leafhopper, mitochondrial genome, phylogeny

## Abstract

In this study, the complete mitochondrial genome of the leafhopper species *Empoascanara dwalata* (Hemiptera: Cicadellidae: Typhlocybinae) was sequenced and annotated, and the sequence is available in the GenBank with accession number: MT350235. The circular mitogenome with 15,271 bp in length, including 13 protein-coding genes (PCGs), 22 tRNA genes, two rRNA genes, and one AT-rich region. The nucleotide composition is as follows: A (41.7%), T (34.7%), G (10.6%), and C (13.0%). 11 PCGs have ATN as the start codon, except for *atp8* and *nad5* genes have TTG. The conventional termination codons (TAA or TAG) occur in 11 PCGs, while *cox2* and *nad5* use incomplete codon (T––) as termination codon. Phylogenetic analysis using 13 PCGs showed that *E. dwalata* was clustered with three other Typhlocybine species, which was consistent with the conventional classification.

At present, the subfamily Typhlocybinae has nearly 5000 species worldwide and is the second largest subfamily of Cicadellidae (Nielson and Knight 2000). The body size is quite small, usually between 2 and 4 mm, and distributed in all Zoogeographical regions. *Empoascanara dwalata* Dworakowska 1971 belongs to the tribe Erythroneurini with *Empoascanara prima* Distant, 1918 as its type species (Dworakowska 1971, 1980). Leafhoppers usually feed on trees and shrubs, and are harmful to woody and herbaceous plants through sucking, spawning, and virus transmission (Du et al. 2017). In this study, all the samples examined were collected from Maolan, Guizhou Province, China. The whole body was preserved in ethanol and deposited in the insect specimen room of Guizhou Normal University with an accession number GZNU-ELS-2019013. The total DNA is extracted from the entire body without the abdomen.

The circular mitochondrial genome of *E. dwalata* is 15,271 bp in size (GenBank no. MT350235), contains 37 typical mitochondrial genes (13 protein-coding genes, 22 tRNA genes, and two rRNA genes) and an AT-rich region. The gene order and orientation of *E. dwalata* were identical to other Hemiptera species. The basic component values of the whole mitogenome are highly A + T biased (A: 41.7%; T: 34.7%, G: 10.6%, C: 13.0%), the total A + T content is 76.4%, AT-skew and GC-skew it is 0.091 and −0.100 respectively. 23 genes were oriented on the majority strand (N-strand), whereas the others were transcribed on the minority strand (J-strand). The mitogenome of the leafhopper species *E. dwalata* has a total of 23 bp intergenic space, which is made up of 7 regions in the range from 1 to 23 bp. The largest intergenic spacer sequence of 9 bp is located between *trnQ* and *trnM*. 12 genes were found to overlap, with a total length of 37 bp, and the longest 8 bp overlap between *trnW* and *trnC*. All 13 PCGs have ATN as the start codon, except that *atp8* and *nad5* genes have TTG. Conventional stop codons (TAA or TAG) appear in 11 PCGs, while *cox2* and *nad5* use incomplete codons (a single T––) as stop codon. All tRNA genes are identified by ARWEN version 1.2 software. The *rrnL* gene is 1184 bp in size and is located between *tRNL2* and *tRNV*. The *rrnS* gene is 729 bp in length and is located after *tRNV*.

The nucleotides sequences of 13 PCGs of *E. dwalata* and 15 reference sequences from other species in family Cicadellidae were used to construct phylogenetic relationship (Figure 1). Maximum-Likelihood (ML) method was used through MEGA 6.06, which constructed with the IQ-TREE using an ultrafast bootstrap approximation approach with 10,000 replicates (Nguyen et al. 2015). The result showed that *E. dwalata* and other three species of subfamily Typhlocybinae were clustered into one clade, which was separated from other subfamilies, and consistent with the conventional classification. Therefore, the molecular data obtained in this study will facilitate future studies on the identification, population genetics, and evolution of the subfamily Typhlocybinae.

**Figure 1.**
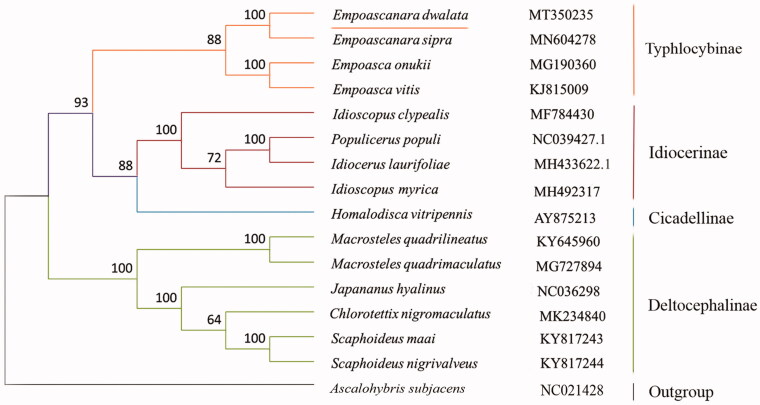
Phylogenetic tree showing the relationship between *E. dwalata* and 14 other leafhoppers in inner group based on Maximum-Likelihood method.

## Data Availability

The authors confirm that the data supporting the results of this study can be obtained from the corresponding author, upon reasonable request, or openly available in NCBI GenBank database at https://www.ncbi.nlm.nih.gov with the accession number is MT350235.
